# The Light Chain Domain and Especially the C-Terminus of Receptor-Binding Domain of the Botulinum Neurotoxin (BoNT) Are the Hotspots for Amino Acid Variability and Toxin Type Diversity

**DOI:** 10.3390/genes13101915

**Published:** 2022-10-21

**Authors:** Renmao Tian, Melissa Widel, Behzad Imanian

**Affiliations:** 1 Institute for Food Safety and Health, Illinois Institute of Technology, Bedford Park, IL 60501, USA; 2 Food Science and Nutrition Department, Illinois Institute of Technology, Chicago, IL 60616, USA

**Keywords:** botulinum neurotoxins, *Clostridium botulinum*, gene diversity, receptor-binding domain

## Abstract

Botulinum neurotoxins (BoNT) are the most potent toxins in the world. They are produced by a few dozens of strains within several clostridial species. The toxin that they produce can cause botulism, a flaccid paralysis in humans and other animals. With seven established serologically different types and over 40 subtypes, BoNTs are among the most diverse known toxins. The toxin, its structure, its function and its physiological effects on the neural cell and animal hosts along with its diversity have been the subjects of numerous studies. However, many gaps remain in our knowledge about the BoNT toxin and the species that produce them. One of these gaps involves the distribution and extent of variability along the full length of the gene and the protein as well as its domains and subdomains. In this study, we performed an extensive analysis of all of the available 143 unique BoNT-encoding genes and their products, and we investigated their diversity and evolution. Our results indicate that while the nucleotide variability is almost uniformly distributed along the entire length of the gene, the amino acid variability is not. We found that most of the differences were concentrated along the protein’s light chain (LC) domain and especially, the C-terminus of the receptor-binding domain (H_CC_). These two regions of the protein are thus identified as the main source of the toxin type differentiation, and consequently, this toxin’s versatility to bind different receptors and their isoforms and act upon different substrates, thus infecting different hosts.

## 1. Introduction

Toxins are organic or naturally occurring poisons, produced by a variety of organisms including bacteria in the form of peptides, single or conjugated proteins with adverse effects on the target cells and organisms. More than any other bacterial class, Clostridia, with over a dozen toxin-producing species especially within the Genus *Clostridium*, makes the highest and the most diverse number of toxins including alpha, beta, gamma, delta, epsilon, iota, zeta, eta, theta, TcdA and TcdB, tetanus and botulinum [1,2]. Each of these toxins is specialized in targeting a specific type of cell or tissue such as red blood cells, the liver or the nervous system in a wide ranging group of animals such as waterfowl, wild birds, cattle, horses, poultry, mink and humans [3].

A subset of the above-mentioned toxins are bacterial neurotoxins that can damage or destruct nerve cells/tissues. Neurotoxins often function through inhibiting the neuron control through disrupting either the ion concentrations across the cell membrane or the communication between the neurons across a synapse [4,5]. The common effects of neurotoxin exposure in humans include widespread central nervous system damage that results in epilepsy, dementia [6], intellectual disability [7] or persistent memory impairments [8]. Different neurotoxins have different potencies, and the botulinum neurotoxin (BoNT) is the most potent/lethal known toxin, produced by several Gram-positive, anaerobic and spore-forming bacteria, mostly in *Clostridium* genus including *C. botulinum*, *C. baratii*, *C. butyricum* and *C. argentinense* [9,10,11,12].

In vivo, the toxic BoNT proteins, like other neurotoxins, form a toxin complex (TC) with a few non-toxic proteins [13], whose genes are in a cluster adjacent to the *bont* gene, located in the bacterial chromosome or extrachromosomal elements such as plasmids. These accessory genes invariably include a non-toxin/non-hemagglutinin (*ntnh*) gene along with either hemagglutinin (*ha*) genes (*ha70*, *ha17* and *ha33*) in some genomes or *orfX* genes (*orfX1*, *orfX2* and *orfX3*) in others. While little is known about the functions of ORFX1-3, a few studies suggest that the products of the *ha* genes are involved in providing help for docking of the BoNT protein to the receptors, found on the lumen of the small intestine [14] and also for the protection for the BoNT protein in its journey from the gastrointestinal tract to the circulatory system [15,16].

The BoNT protein is initially produced as a single soluble polypeptide chain, weighing 150 kDa. This precursor protein is not toxic to the neural tissues, until later, when it is cleaved by a protease that is either produced by the bacterium or made within the target cell/tissue itself [17]. The cleaving of the precursor protein generates two polypeptide chains that are linked by a functionally critical disulfide bond, and it results in the activation of the neurotoxin. One of the polypeptide chains, the light chain (LC, 50 kDa), has a zinc endopeptidase activity and it is, thus, the catalytic domain [18,19,20]. The function of the LC is to cleave a target protein in the neural cells such as the Soluble N-ethylmaleimide-Sensitive Factor Attachment Proteins (SNAP) Receptor (SNARE), with three SNARE proteins identified so far: Vesicle-Associated Membrane Protein (VAMP)/synaptobrevin, SNAP-25 and syntaxin [20]. The other polypeptide, the heavy chain (HC, 100 kDa), is composed of two functional domains: an N-terminus (H_N_) translocation domain and a C-terminus (H_C_) receptor-binding domain [19,21,22]. The H_C_ domain of the heavy chain can be further divided into two subdomains: the N-terminus (H_CN_) and the C-terminus (H_CC_). The three domains of BoNT work together to intoxicate the host. The specificity of a neurotoxin is ensured through its receptor-binding domain that recognizes specific receptor(s) in/on and delivers their effects to the specific neural cells. In the case of H_C_, the specific recognition of neuronal cells occurs by a double anchorage to the receptors: first, to a polysialoganglioside (PSG) receptor, and then, this is followed by binding to a protein like synaptotagmin (Syt) (e.g., target of BoNT/B and BoNT/G) in two known isoforms (I–II) or in a Synaptic Vesicle protein (SV) receptor (e.g., target for BoNT/A and BoNT/E), with three identified isoforms (A-C) [23,24,25,26,27]. The special double receptor mechanism guarantees a specific binding to no other target except for the neural cell. Then, BoNT is internalized through its H_N_ translocation domain and the clathrin–dynamin-mediated endocytosis of the recycling neurosecretion vesicles [28]. The endocytosis of BoNT is followed by the acidification of the lumen of the vesicle and the conformational change of BoNT. Finally, the LC zinc endopeptidase cleaves the SNARE proteins, which are involved in neurotransmitter release, thus, blocking the acetylcholine release, leading to a flaccid paralysis of the inflicted organism [20,29].

BoNTs are extraordinarily diverse and so are the species that produce them. Many studies have investigated the diversity of the BoNT-producing bacteria, employing serological, physiological, biochemical, structural and genetic and genomic methods and analyses. For a long time, producing the botulinum neurotoxin was the only criterion to classify a species as *C. botulinum*. Based on several phenotypic and biochemical differences, four different groups (I-IV) were recognized within the BoNT-producing ‘*C. botulinum*’ that are now, in the light of recent molecular interrogations, acknowledged to be at least four different taxonomic species [10]. As for the BoNT diversity, there are currently seven or possibly eight serologically different established types of proteins/toxins, designated with letters A–G (amino acid differences ranging from 37.2% to 69.6%) [30], with over 40 BoNT subtypes (e.g., A1, B1, E12), and also chimeric forms with a BoNT type appearing in combinatory forms with another BoNT type or types, usually leading to the production of more than one toxin type and often in different ratios (e.g., BoNT/CD, /DC, /FA, /A2F4, /A2F5, /A2B6, /B5A4) [10,15,31,32]. Recent genetic studies have uncovered new types of BoNT, for example, BoNT/H [33], later characterized as a chimeric BoNT/FA [20] and as BoNT/HA [34], and BoNT-like proteins, BoNT/Wo, BoNT/J [35] and BoNT/X [36]. Types A, B, E and F can cause botulism in humans [37], while BoNTs C and D intoxicate animals including birds and mammals. [3,38]. Not much is known about the effects of BoNT/G or the newly discovered BoNT types (H, J and X) on humans or animals. Interestingly, BoNTs are found to have also valuable cosmetic and medical uses [39,40].

Beyond satisfying the academic curiosity and interest, studying the BoNT diversity provides real-life, practical, medical and health benefits since the big or small differences at the nucleotide/amino acid levels are not trivial. These differences determine the toxin types and subtypes, influencing the final folding of the proteins, and thus, their functions and efficiencies at every step from the formation of TC and receptor binding to the BoNT internalization within the neural cell and the endopeptidase activity of LC domain. They can, therefore, affect intoxication and the kind and intensity of botulism symptoms in vivo among other things [20].

A mplified fragment length polymorphism (AFLP) analyses, gene (e.g., *16S rRNA*, *bont*) and whole genome sequencing in conjunction with various phylogenetic/phylogenomic and other molecular techniques and analyses have been employed to tackle the important question of the diversity of BoNT-producing species and strains, *bont* genes and BoNT proteins. These approaches, strengthened by ecological and biogeographical studies, have begun to (1) help researchers to revise and improve the confusing historical taxonomy and evolutionary relationships between the BoNT-producing species/strains; (2) identify the location of the *bont* gene and the *bont* gene clusters (chromosomal, extrachromosomal); (3) unravel the *bont* gene and protein evolution; (4) discover new BoNT subtypes and occasionally new types; (5) provide new insights into BoNT pathogenesis [41,42,43,44,45]. Despite these excellent studies, many gaps remain in our knowledge about the diversity and evolution of *bont* genes and proteins. In order to address one of these gaps and to provide a more granular insight into the BoNT diversity, we investigated the distribution of this diversity among different domains and subdomains of all the available BoNT proteins, including the within types and between types and spatially along the 3D structure of BoNT, with the null assumption that the amino acid variations along different domains/subdomains have a uniform distribution. The results of our study clearly show that it is not the case, and the LC domain, and especially, the H_CC_ subdomain of BoNT are the hotspots and the sites for the highest number of amino acid variations.

## 2. Materials and Methods

### 2.1. Collection of Bont Gene Sequences

Sequences of the genes for botulinum neurotoxin (*bont*) were collected through a custom pipeline. Seed sequences of the genes were obtained (81 sequences in total) from NCBI RefSeq database https://www.ncbi.nlm.nih.gov/protein (accessed on 15 November 2021) by searching the protein names. The sequences were further processed to manually remove the incorrectly annotated sequences (e.g., based on the sequence description). The seed sequences were then clustered at 95% identity to acquire representative sequences (28 in total) using CD-HIT (version 4.8.1) [46], which were then used as queries for tBLASTn against NCBI NT database. The output was parsed using the Python package Bio.Blast.NCBIXML to screen for qualified hits with identity > 30% and alignment coverage > 80%. The accession IDs of qualified hits were used to retrieve the nucleotide coding sequences using the Python package Bio.efetch, and the aligned regions were extracted according to the alignment coordinates. The downloaded sequences were then clustered at 100% identity using CD-HIT to remove the duplicates. Because botulinum and tetanus neurotoxin genes share ~30% sequence similarity, the sequences of *bont* with taxonomic assignment of *Clostridium tenani* in the sequence description were removed (and so, 143 sequences remained).

### 2.2. Assignment of Toxin Types

The above seed BoNT protein sequences (81) were entered into a custom database, against which the collected nucleotide coding sequences (143) were searched using BLASTx (version 2.12.0) [47] with a query coverage cutoff of 90% and an E value cutoff of 10^-5^. The BLASTx output was then parsed, and the best hits with an identity > 90% were selected for the toxin type assignment. For the subtype analysis, the protein sequences were clustered using CD-HIT (version 4.8.1) at 97% identity, and each cluster was assigned a unique cluster ID of its own type. We then investigated the sequence variation within each cluster. Note that the sequences within a type do not exactly correspond to the subtypes defined in other studies, which may include sequences with as little as 1.5% dissimilarity to as high as 36.2% sequence variation [10,32,48,49].

### 2.3. Alignment and Sequence Diversity Analysis

All of the nucleotide coding sequences were first translated to protein sequences using the Python package Bio.SeqIO with the genetic codon table 11. Any pseudogenes with premature stop codon were removed. The protein sequences were then aligned using MUSCLE (version 3.8.1551) [50]. The nucleotide coding sequences were also aligned using PAL2NAL (version 14) [51] with the protein sequence alignment being used as a template. The protein or nucleotide sequence multiple alignment output was imported with the Python package Bio.AlignIO, and it was converted into a 2D array with the Python package Numpy. In order to construct a sequence diversity index, we counted the number of unique amino acids or nucleotides of each column in the protein and gene multiple alignment files, respectively. To demonstrate the sequence variation in each domain, the sequence diversity indexes of all of the positions were calculated and mapped onto the nucleotide sequence of BoNT/A of *C. botulinum* 62A; a commonly used reference in *C. botulinum* studies. The same was performed for each type of BoNT gene to investigate the sequence variation profiles.

### 2.4. Visualizing BoNT Sequence Variation on a BoNT 3D Structure

In order to visualize the sequence variation on a 3D structure of a BoNT protein, we mapped the diversity index at each position onto PDB data. A reference protein crystal structure PDB data (3BTA, serotype A) which was highly similar (>99.9%) to the reference protein sequences in the diversity analysis was downloaded from the Protein Databank (PDB) database. The amino acids and corresponding coordinates were retrieved from the PDB file using the Python package Bio.PDB. The retrieved protein sequences were aligned to the reference protein sequence in the diversity analysis, and then, the diversity indexes were mapped onto each amino acid position in the PDB data by replacing the B factor value with the corresponding diversity index. The sequence diversity was then visualized by importing the revised PDB file into PyMOL (version 2.5.2) (Schrödinger, LLC, New York, NY, USA) and executing ‘spectrum b, blue_white_red’ command. The same procedure was performed for each toxin type separately to demonstrate within-type sequence variation on the 3D structure for each type.

### 2.5. Visualizing the BoNT Amino Acids Interacting with Ganglioside, SV2 and Monoclonal Antibody (mAb) CR1 on a Reference 3D Structure

The PDB data of the reference BoNT protein crystal structure combined with ganglioside, SV2 and monoclonal antibody (mAb) were searched and downloaded from the PDB database. Amino acids from the BoNT interacting with the ligands were identified using iCn3D [52] with the function icn3dnode. The protein sequence was aligned with the one from the analysis of sequence variation in 3D structure and the corresponding interacting amino acids were labelled in the visualization.

## 3. Results

### 3.1. Gene Sequence Collection

In total, nucleotide coding sequences of 143 unique *bont* genes were collected from the NCBI NT database. One of these sequences was identified as a pseudogene due to it having a premature stop codon, and it was removed from the analysis. We were able to assign a toxin type to 131 of the collected sequences (Figure S1). There were four dominant types: A (29 genes), B (34 genes), E (35 genes) and F (17 genes). Due to the low number of retrieved sequences (<7), the types C, D, G, H and X were not considered for further analysis.

### 3.2. The LC Domain and Especially the C-Terminus of the Receptor-Binding Domain (H_CC_) of BoNT Contain More Amino Acid Diversity Than Other Domains Do

**Figure 1:**
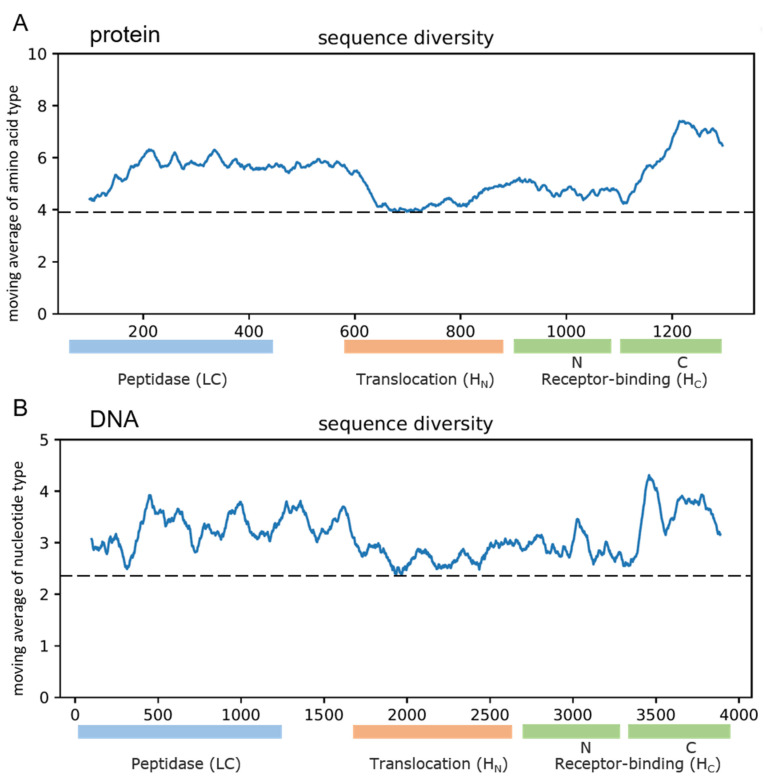
The amino acid and nucleotide diversity distribution of different regions of botulinum neurotoxin (**A**) protein and (**B**) gene, respectively. The diversity index (produced by counting the number of unique amino acids or nucleotides in each column in the multiple alignment files) of all of the positions were calculated using *C. botulinum* 62A BoNT gene as a reference. The moving average (*n* = 100) of the diversity index was used to generate an overview of the sequence variation. The dashed black horizontal lines demarcate the lowest diversity value in the graph and are used only as visual aid.

Our nucleotide and amino acid diversity index analyses of the *bont* genes and the BoNT proteins revealed that the distribution of amino acid variations is not uniform along the protein and its various domains and subdomains. We found that the light chain domain (LC, 4–409), and especially, the C-terminus of the receptor-binding domain (Hcc, 1088–1293 aa) contained more amino acid diversity, 24% and 45% more than that in the HN domain, respectively, with the peak occurring along the end of the H_CC_ subdomain (Figure 1).

### 3.3. Inter-Type Amino Acid Variation Was Much Higher Than Intra-Type Variation in BoNT Proteins

**Figure 2:**
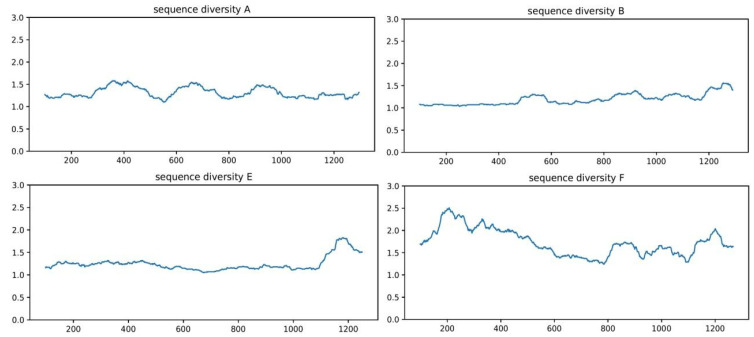
Protein sequence diversity within each type of BoNT. Protein sequences of each type were aligned and the diversity index (produced by counting the number of unique amino acids or nucleotides in each column in the multiple alignment files) of all of the positions were calculated and mapped onto a representative BoNT gene of each type. The moving average (*n* = 100) of the diversity index was shown for an overview of the sequence variation.

We further compared the protein sequences within each BoNT type, for which a sufficient number of sequences were available (A, B, E and F). A close inspection of the diversity index within each BoNT type revealed that in each position in the alignment file for types A, B and E, there were on average only one to two amino acid types or gaps. The highest variability was found within type F (2.5 on average for some positions, Figure 2). We also conducted similar calculations at the subtype level for the clusters within each type, and we found that the protein sequences within each cluster were even more conserved, with the sequence diversity index being < 1.3 in all of the subtypes (Figure S2). By contrast and not surprisingly, the between-types comparisons showed more amino acid variability for the BoNT proteins (with the diversity index value > 3.9 on moving average), reaching a maximum of eight different amino acids per position along the H_CC_ domain (Figure 1A). To recap, the protein sequences were found to be highly conserved within each BoNT type, and they were even more conserved within the subtypes, and highly variable between the types, with the highest amino acid variability being observed along the H_CC_ subdomain.

### 3.4. The Sequence Variations Are Mainly Concentrated at the Surface of the Protein’s 3D Structure Where the LC Domain and HCC Subdomain Are Located

**Figure 3:**
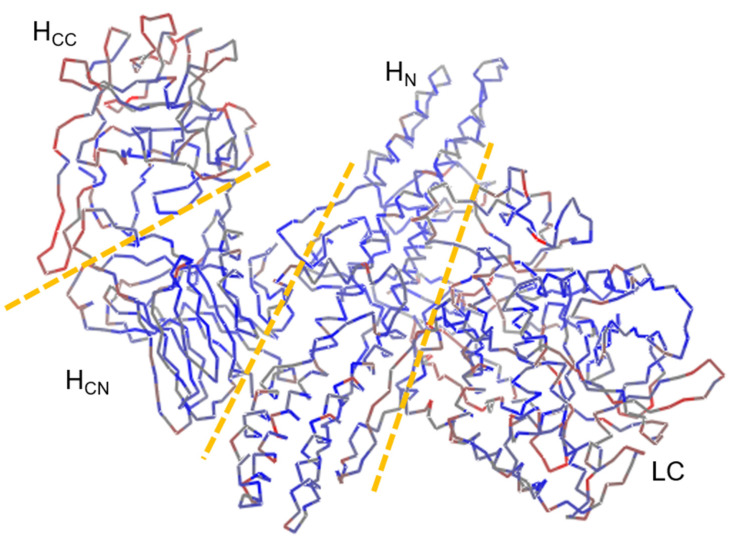
Amino acid sequence diversity of BoNT proteins mapped on its 3D structure (3BTA, serotype A). The diversity index, the actual counts of the number of unique amino acids or nucleotides of each column in the multiple alignment files, of all the positions were mapped on the 3D structure of a reference gene, 3BTA, downloaded from the PDB database. The diversity index is indicated by different shades of a color from blue (low) to medium (grey) to red (high). LC: Light chain. H_N_: Heavy chain N-terminus. H_CN_: N-terminus of heavy chain C-terminus. H_CC_: C-terminus of heavy chain C-terminus.

In order to examine the spatial location of the highly variable amino acids, we mapped the sequence diversity profile (the actual diversity index, rather than the moving average values) on the 3D structure of the BoNT protein (3BTA, serotype A). We found that the light chain (LC, peptidase domain) and the H_CC_ region of the receptor-binding domain had more amino acid diversity than the other domains and regions of the protein did. These two domains are located near the ends of the protein (N-terminus and C-terminus, respectively). In contrast, the N-terminus of the receptor-binding domain (H_CN_) and the translocation domain (H_N_) that harbored less amino acid diversity were located near the core of the folded protein, away from the BoNT protein’s termini. In addition, within the H_CC_ sub-domain, the highly variable amino acids were found to be located on the exterior or surface of the folded protein, while the more conserved positions were embedded in the interior or core of the domain (Figure 3). In summary, the amino acids on the surface or exterior of the folded BoNT protein showed more diversity than those that are positioned interiorly with the maximum diversity values being observed along the surface amino acids of the LC domain, and especially, on the H_CC_ sub-domain.

**Figure 4:**
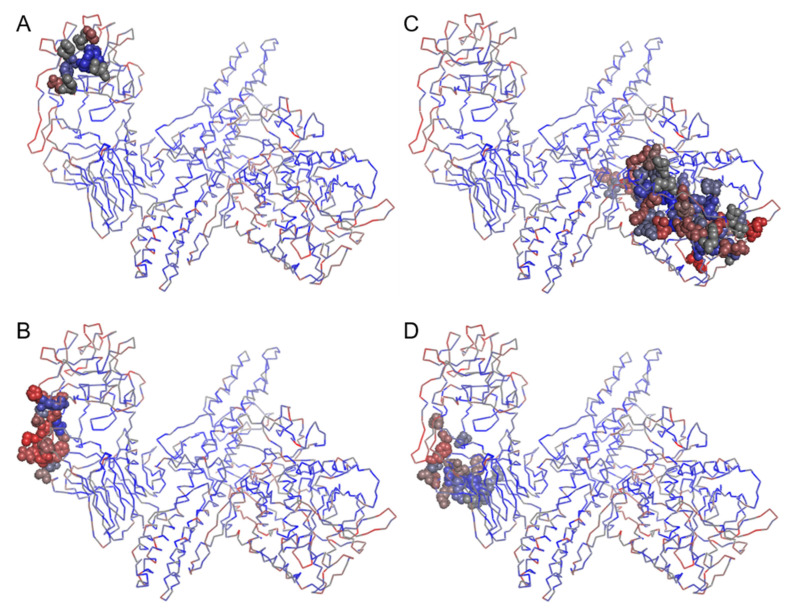
Three-dimensional structure of BoNT protein and the interacting sites with (**A**) ganglioside, (**B**) SV2, (**C**) SNAP-25 substrate and (**D**) monoclonal antibody CR1 against BoNT toxin. The diversity index of each position were mapped onto the 3D structure indicted by the color (from blue to grey to red, low to medium to high). The interacting amino acids were highlighted as spheres with color indicating the diversity index.

In order to further explore the function of the highly diverse amino acids of the H_CC_ sub-domain of the receptor-binding domain of the BoNT protein, we identified the sites that interacted with the receptors, a ganglioside and a synaptic vesicle (SV2), and also with a SNAP-25 substrate and a mAb CR1 against the BoNT toxin. Our examinations indicated that the highly variable sites of H_CC_ were mostly made of the same residues that interacted with the two receptors (Figure 4A,B). The different isoforms of the ganglioside had highly consistent interacting sites in BoNT (Figure S3). Interestingly, the interacting sites with the mAb CR1 against the toxin and those interacting with the SV2 receptor clearly showed a large overlap (Figure 4B,D), hinting at the possible modes of function for the antitoxin quality of mAb CR1 by competing with or blocking the sites that bind the SV2 receptor.

## 4. Discussion

With a few exceptions, all of the BoNT-producing clostridia were traditionally classified as *C. botulinum*, whose members then were categorized into several groups based on a few physiological characteristics (e.g., proteolytic differences) [53,54], thus leading to a great deal of confusion about the taxonomy, phylogeny and diversity of this class of bacteria. Recent molecular studies have begun to address and resolve this confusion. Characterizing the diversity of the neurotoxin genes (*bont*) and their products (BoNT) is particularly important because the differences between the BoNT types and the subtypes determine, or at least influence, the functional speed and efficiency of the toxin, the class or type of receptors and the hosts, intoxication, botulism symptoms, and so on [20]. Many of the previous studies have focused on characterizing and enumerating the toxin types/subtypes and cataloguing and quantifying the sequence similarities and differences. The diversity of the *bont* genes and their products have also been explored by employing a phylogenetic analysis of the *bont* genes, the BoNT proteins with or without the toxin accessory genes and in the *bont*-encoding clostridial species/strains [12,55]. However, few studies have focused on the distribution of the nucleotide and amino acid diversity along the entire *bont* gene, its product and its domains/subdomains [10] across the primary sequence as well as spatially along the 3D structure of the folded protein in the clostridial species/strains.

Here, we examined this distribution, and we found that the amino acid variations generally increased from the within-subtypes (Figure S2) to the within-types (Figure 2), and from there to the between-types of toxins (Figure 1), where the variations peaked. These variations at different levels might provide clues as to why different types or subtypes of the toxin function differently with different degrees of efficiencies. As an example, the LC domain of the BoNT/F contains more variations than the same domain does in the other BoNT types (Figure 2), and this could provide one possible explanation for the differential catalytic activities of the LC domain that has been reported in different BoNT/F subtypes [32].

We also found that the nucleotide variations had nearly uniform distribution along the entire length of all of the available *bont* genes. However, the amino acid variations were not uniform along the length of the protein (BoNT) and its various domains and subdomains. This implies that while all of the types of mutations might have occurred uniformly along the gene sequence, the non-synonymous or missense mutations that change the encoded amino acid had not. In fact, the LC domain and especially the C-terminus of the receptor-binding subdomain (H_CC_) in the BoNT proteins harbored more diversity (contained more amino acid types per position) than the other domains/subdomains did. In other words, the LC and particularly H_CC_ were the main contributors to the total diversity found in the BoNT proteins, and thus, they are mainly responsible for BoNT type differentiation. Further studies are needed to investigate whether these two domains are under selective pressure, and diversifying these two domains is accompanied by any improved fitness, and perhaps, this may contribute to the speciation of the clostridial species.

Mapping the amino acid type diversity of the BoNT proteins on a 3D reference structure provided a quick visual reference for the diversity of the BoNT proteins, demonstrating the same pattern, but here spatially: there was more amino acid diversity within the LC domain, and especially, in the H_CC_ subdomain of the protein (Figure 3). The expected consequences of this diversity especially in these two regions of the protein have already been documented. For example, it has been shown that the LC of different BoNTs in different species/strains cleave different SNARE proteins with many cleavage sites having been already identified. For example, BoNT/B, BoNT/D, BoNT/F and BoNT/G cut VAMP, while BoNT/A and BoNT/E cleave SNAP-25, and BoNT/C cleaves SNAP-25 as well as syntaxin (Stx) [20]. More importantly, we also found, and here we show, that many of the amino acids within the receptor-binding domain that interacted with the ganglioside, the SV2 receptors and the SNAP-25 substrate on the target cells were among the highly variable amino acids (Figure 4A–C). It has been demonstrated that BoNT acts on different hosts and its targets have usually more than one isoform: the ganglioside receptors have several (e.g., GM1, GD1a, GD1b and GT1b) [31,56]; the synaptotagmin (SytI-II) has two known isoforms; and the SV2 receptors have at least three (SV2A-C) [31,57]. Our results once more show and underline that the functional diversity of the BoNT proteins can be linked, at least in part, to the observed nucleotide variability of the *bont* genes and the amino acid type diversity of their products in different clostridial species/strains. This underlying diversity is at the heart of BoNT’s versatility in infecting different hosts, targeting different receptors, acting on different substrates with different speed and efficiencies and causing different symptoms with different intensities. Studies in avian and mammalian botulism, for example, have confirmed that BoNT/C and BoNT/D are specific to animal rather than human hosts [3,38], whereas BoNT/A, BoNT/B, BoNT/E and BoNT/F cause botulism in human rather than animal hosts. The sequence and structure of SV2 and its various isoforms in animals are different enough from those in humans so that the BoNT/C and BoNT/D could bind the receptor(s) in the target cell and/or cleave the substrate in animals, but rarely in humans.

Some of the recent studies have shed light on the possible underlying mechanisms behind this extraordinary diversity. In addition to the slow random mutations, researchers have discovered extensive evidence of multiple horizontal gene transfer (HGT) events of the *bont* gene or the *bont* gene cluster between the closely or distantly related strains and species and the footprints of homologous recombination [42,54,57]. While it is clear that BoNT diversity plays an important role in the collective versatility of the toxin in binding a variety of receptors that are found in a number of different hosts and in the success of the BoNT-producing species, it is not clear whether there is a selective pressure driving the diversification of BoNT and its domains and subdomains. It is conceivable that host specialization by the Clostridial species is one of the possible consequences of this diversification, a specialization that could lessen the costly competitions between the closely related bacterial species in the same environment. However, further studies are needed to validate this speculation.

## Data Availability

Not applicable.

## Supplementary Materials

The following supporting information can be downloaded at: https://www.mdpi.com/article/10.3390/genes13101915/s1. Figure S1: Number of bont genes collected for each type. The types C, D, G, H and X had <7 sequences and were thus not considered for further comparison analysis; Figure S2: The diversity index at subtype level. Subtypes were generated by protein sequence clustering at 97%. (A) shows the number of protein sequences of each subtype. (B) shows the diversity index, counting the number of unique amino acids of each column in the multiple alignment, of each subtype. The moving average (*n* = 100) of the diversity index were shown for an overview of the sequence variation; Figure S3: Interacting sites of BoNT with ganglioside receptors. The highlighted spheres corresponds to the interacting sites with ganglioside (A) GD1a (in 5PTC), (B) GT1B (in 2VU9), (C) GD1a (in 7QPT). The interacting sites were mapped onto the BoNT structure from PDB database (3BTA). The amino acid residue numbers were displayed.
