# Supplementary material for: The Light Chain Domain and Especially the C-Terminus of Receptor-Binding Domain of the Botulinum Neurotoxin (BoNT) Are the Hotspots for Amino Acid Variability and Toxin Type Diversity
Source: Genes (Basel). 2022 Oct 21;13(10):1915. doi: 10.3390/genes13101915 (PMC9601653; doi:10.3390/genes13101915)
Supplement: Supplementary file 1 [file genes-13-01915-s001.zip › genes-1964707-supplementary.pdf]

Supplementary Materials

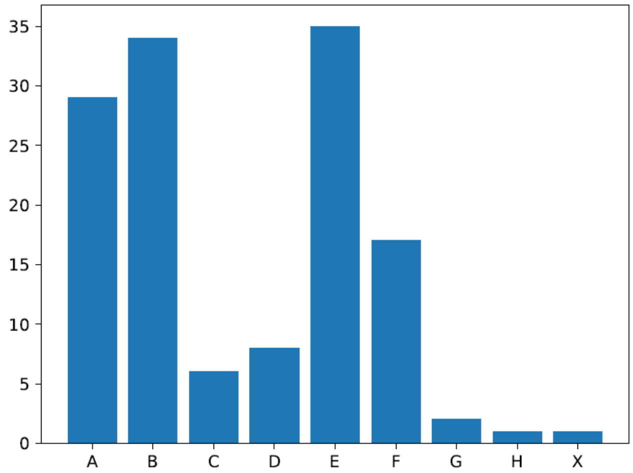

Figure S1. Number of *bont* genes collected for each type. The types C, D, G, H and X had < 7 sequences and were thus not considered for further comparison analysis.

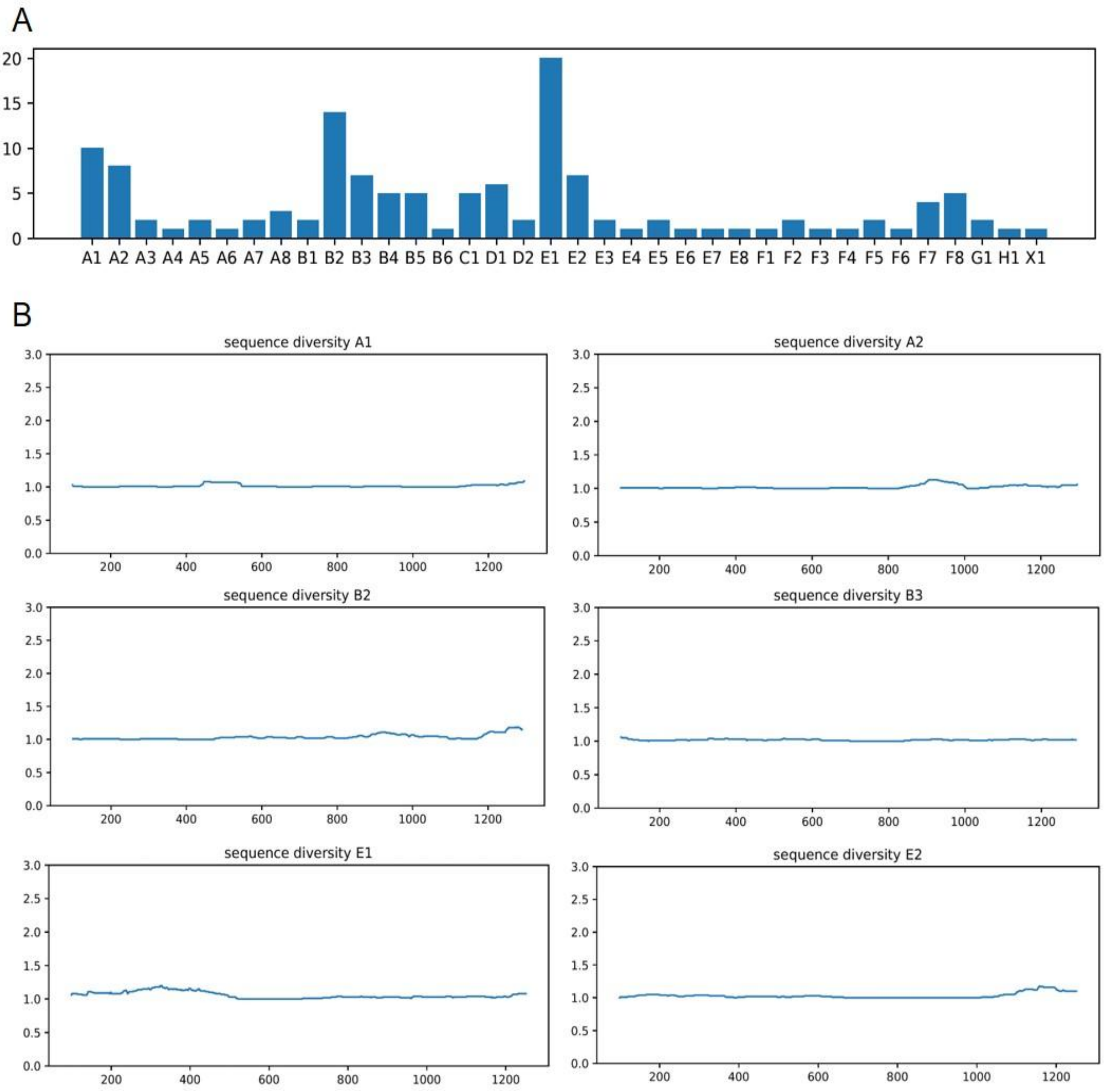

Figure S2. The diversity index at subtype level. Subtypes were generated by protein sequence clustering at 97%. (A) shows the number of protein sequences of each subtype. (B) shows the diversity index, counting the

number of unique amino acids of each column in the multiple alignment, of each subtype. The moving average (n = 100) of the diversity index were shown for an overview of the sequence variation.

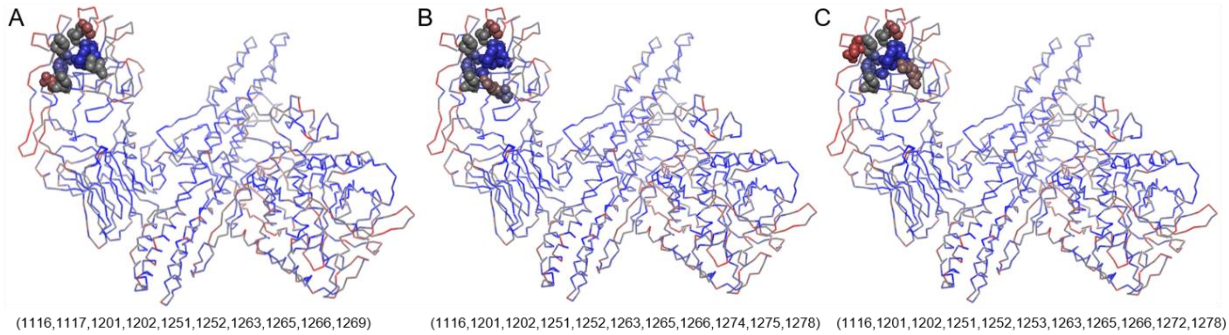

Figure S3. Interacting sites of BoNT with ganglioside receptors. The highlighted spheres corresponds to the interacting sites with ganglioside (A) GD1a (in 5PTC), (B) GT1B (in 2VU9), (C) GD1a (in 7QPT). The interacting sites were mapped onto the BoNT structure from PDB database (3BTA). The amino acid residue numbers were displayed.
